# Inflammation in the CNS and PNS: From Molecular Basis to Therapy

**DOI:** 10.3390/ijms24119417

**Published:** 2023-05-29

**Authors:** Savina Apolloni, Nadia D’Ambrosi

**Affiliations:** Department of Biology, University of Rome Tor Vergata, 00133 Rome, Italy

Our understanding of the pathophysiology of the nervous system has advanced significantly in the last few years, but there are still many unanswered questions. The impact of acute and chronic inflammation on neurodegenerative mechanisms is particularly challenging as this response begins as a protective mechanism. Still, it often ends up exacerbating the progression of neurological diseases. Therefore, comprehending the pathways triggered in the inflammatory response and their temporal orchestration could be instrumental in identifying mechanisms and time windows to intervene appropriately to limit and redirect this process into an advantageous event. Inflammation occurs by recruiting different players in the central nervous system (CNS) and peripheral nervous system (PNS) and encompassing distinct cellular and molecular mechanisms. For example, blood cell infiltration and blood–brain barrier (BBB) disruption often characterize peripheral inflammation, while the central involvement of resident immune cells as microglia is a distinct tract of CNS-related responses [1]. Moreover, the interaction between peripheral tissues and central areas of the nervous system, particularly the propagation of the inflammatory axis to CNS in certain circumstances, constitutes an additional element of complexity [2]. The definition of common determinants and distinct mechanisms in these contexts may therefore be helpful to simplify the elaborate cellular and molecular landscape of neurodegenerations and neuropathies.

In this Special Issue entitled “Inflammation in the CNS and PNS: From Molecular Basis to Therapy”, two original and five review articles were published, aimed to define the role of inflammation in human diseases affecting the central and peripheral nervous system, including Friedreich’s, Huntington’s disease, multiple sclerosis, and peripheral neuropathies.

The article by Pyka-Fościak and colleagues [3] sheds light on the complex interactions between adhesion molecules and leukocyte migration across the blood–brain barrier (BBB) in the murine model of multiple sclerosis (MS), experimental autoimmune encephalomyelitis (EAE). The study examines the expression of various adhesion molecules (such as ICAM-1 and LFA-1) in different phases of EAE, demonstrating that they display discrete stages of increased expression corresponding to the onset and peak of the disease. Moreover, they showed that anti-VLA-4 monoclonal antibodies decrease the expression of adhesion molecules, suppress inflammatory pathways, delay the clinical disease onset, and reduce clinical scores in the subsequent phases of EAE, demonstrating the potential for leukocyte migration-related therapies in MS. Indeed, the issue regarding the BBB as a vulnerable site in chronic inflammation was likewise the subject of the study by Da Rocha and colleagues [4]. The study found that pioglitazone, a peroxisome proliferator-activated receptor γ agonist, effectively inhibited altered pathways engaged by plasma from inflammatory bowel disease (IBD) patients and related to BBB structure and integrity. The results suggest, therefore, that pioglitazone may be a promising treatment option for inflammatory conditions impacting the CNS. Moreover, this paper highlights the importance of investigating the effects of chronic peripheral inflammation on BBB integrity and on the propagation of injury to the CNS. This last issue is the focus of the review article written by Gao and colleagues [5]. In their analysis of the current literature, the authors discuss how Rheumatoid Arthritis (RA), a chronic, inflammatory disorder affecting the joints, is associated with neuropsychiatric comorbidities, increasing the risk of neurodegenerative diseases. Their overview highlights the importance of exploring the complex interactions between the immune system and the CNS and PNS in the development and progression of autoimmune diseases. In their review article, Wu and colleagues [6] provide insight into a recent treatment method for carpal tunnel syndrome and other peripheral entrapment neuropathies. A non-surgical treatment consisting of perineural injection with 5% dextrose water demonstrated effectiveness by downregulating capsaicin-sensitive receptors (transient receptor potential vanilloid receptor-1, TRPV1) and, consequently, decreasing inflammation and neuropathic pain, with possible neurodegenerative effects.

To trace common determinants in the pathophysiology of the nervous system, Tan and colleagues discuss the role of type I Interferons (IFNs) in different neurological contexts. They evidence that type I IFNs play an active function in regulating cognition, aging, depression, neurodegenerative diseases and pain through the modulation of the cross-talk between neurons and glia. The authors also discuss the role of type I IFNs in long-haul COVID-associated neurological disorders [7].

Lastly, the issue regarding neuroinflammation in Huntington’s disease (HD) and Friedreich’s ataxia (FRDA) is tackled, respectively, by Paldino and Fusco [8] and Apolloni and colleagues [9]. While in other neurodegenerative diseases, such as Alzheimer’s, Parkinson’s, multiple sclerosis, and amyotrophic lateral sclerosis, it is well established that inflammation by resident immune cells of the CNS contributes to initiation or exacerbation of neuron damage, in the case of HD and FRDA this issue has emerged more recently. In the case of HD, in the last decade, there has been an increase in the knowledge of neuroinflammatory pathways in the disease and the potential of targeting the inflammasome and NLRP3 to treat HD [8]. Similarly, in FRDA, recent research has suggested that glial cells may contribute to the pathogenesis of the disease, and that neuroinflammation may play a role in neuropathology. The authors of this review article provide an overview of the evidence linking neuroinflammatory-related mechanisms to FRDA and propose the modulation of glial-related mechanisms as a possible strategy for improving disease features [9]. These studies emphasize the potential of targeting inflammation and glial cells to treat neurodegenerative diseases.

In conclusion, while significant advances have been made in our understanding of the pathophysiology of the nervous system, many questions remain unanswered, particularly regarding the effects of inflammation. Neuroinflammation can have both harmful and beneficial effects on the nervous system, making it a challenging area of study.
